# Exploring stress and coping skills of medical students: a repeated cross-sectional cohort study

**DOI:** 10.5116/ijme.6984.a86a

**Published:** 2026-02-19

**Authors:** David W. Musick, Tracey M. Criss, Mariah J. Rudd, R. Brock Mutcheson, Daniel Harrington, Aubrey L. Knight

**Affiliations:** 1 Department of Internal Medicine, Virginia Tech Carilion School of Medicine, USA; 2 Department of Psychiatry, Virginia Tech Carilion School of Medicine, USA; 3 Department of Obstetrics and Gynecology, Virginia Tech Carilion School of Medicine, USA; 4 Department of Health Systems and Implementation Science, Evaluation and Strategic Analytics, Virginia Tech Carilion School of Medicine, USA; 5 Departments of Internal Medicine and Family Medicine, Virginia Tech Carilion School of Medicine, USA

**Keywords:** Medical student stress, coping strategies, medical education

## Abstract

**Objectives:**

To examine stressors and coping skills as reflected in the student population at a southeastern United States medical school, including identifying key stressors over time and coping mechanisms used.

**Methods:**

Repeated cross-sectional cohort, mixed-methods study conducted between 2016 and 2022 at a four-year medical school program. Participants were students from seven classes, with two classes providing data during each of their four years of medical school. A census sampling approach was used, with survey data collected annually from each class across four years. Two surveys were used:  the Perceived Stress Scale (PSS) and a modified Coping Orientation to Problems Experienced (COPE) Inventory.  Open-text questions captured qualitative responses. Statistical analysis included Welch’s t-tests, Pearson correlations, and Cronbach’s alpha reliability testing. Qualitative data were examined through inductive thematic analysis.

**Results:**

Students reported moderate levels of perceived stress across all four years with fluctuations identified by year of study. There were no statistically significant differences in perceived stress based on student gender; however, qualitative findings identified gender differences related to coping strategies. Thematic analysis of qualitative data revealed three recurring categories of stressors: academic workload, residency application and match pressures, and personal life challenges. Stressors shifted from academic in the pre-clinical years to career concerns during the clinical years.

**Conclusions:**

This study highlights the presence of stress throughout medical school and underscores the importance of adaptive coping strategies and the need for phase-specific interventions to support student well-being. Future research should evaluate the effectiveness of interventions in reducing stress across training stages.

## Introduction

The process of becoming a physician is an inherently stressful experience.  This experience begins before being accepted into medical school, as undergraduate college students intent on careers in medicine must achieve very high levels of performance in comparison to their peers in order to be competitive for acceptance.  And, once enrolled in medical school, students begin an intense education process that lasts from seven to sixteen years, depending on choice of specialty.  Research focused on the experience of medical students has documented significant levels of psychological stress and burnout associated with medical school,^1^^-^^4^ with some studies indicating that medical school can erode students’ natural resilience to stress.^5^^-^^7^ A systematic review^8^ highlighted that medical students experience higher rates of depression and suicidal ideation than their non-medical student peers, emphasizing the urgent need for mental health interventions.

Stress is generally defined as a sense of emotional strain or tension that occurs as a result of circumstances that are demanding in nature.  Stress can be viewed either positively or negatively, resulting in either motivation to reach one’s personal and/or professional goals (eustress) or in less optimal mental health conditions (distress).  Stress can occur because of a sudden, unanticipated event (acute) or be repetitive and last longer periods of time (chronic).  Chronic stress has been shown to impair cognitive function, memory, and problem-solving abilities, which are critical for medical students’ learning and performance;^9^ furthermore, it can negatively affect interpersonal skills, communication, and empathy, which are essential qualities for future healthcare professionals.

A seminal textbook on stress and coping indicates that each individual impacted by a stressful event or situation undergoes a process of “cognitive appraisal” of the situation, which involves an assessment of the event and how one might cope with it; this assessment may be impacted by such factors as personality traits and the nature of the stress itself.  Reactions to stress involve coping mechanisms, which reflect one’s attempts to deal with both the internal and external demands of a given stressful situation.^10^ Medical training may erode students' natural coping mechanisms, making it crucial to understand how they manage stress and what strategies prove effective.^11^

Studies concerning various aspects of physician training and their impact on the eventual development of medical professionalism have been grouped into two broad categories:  environmental factors and personal factors.^12^ In our view, this same framework can be used to examine the four-year medical school experience and how it may be related to student stress.  Studies focused on environmental factors contributing to student stress have examined the effects of the overall medical school learning environment;^12^^,^^13^^-^^14^ a demanding curriculum including associated workload, time pressures and high consequential examinations;^15^ transition periods within medical school, i.e., from the pre-clinical to the clinical phase;^16^ and grading systems used.^17^^-^^18^

Regarding personal factors contributing to medical student stress, some studies have shown that stress can be associated with positive outcomes including academic success, increased confidence, fostering helpful connections with others and the ability to build resilience.^19^^-^^20^ However, studies concerning negative outcomes associated with stress are more plentiful.  Studies on personal factors contributing negatively to student stress have examined specialty choice and career ambition;^21^^-^^22^ burnout;^5^^, ^^23^ self-efficacy and personality traits;^24^ social isolation and a sense of competition;^20^ personal resilience and life satisfaction;^25^ general mental health, anxiety and depressive symptoms;^6^^,^^26^^-^^30^ confrontations with high stress clinical events such as death and grief;^31^ and personal life events.^32^

In combination with a negative learning environment and burnout, studies have found that medical students may also experience additional stressful challenges including imposter syndrome (a phenomenon characterized by feelings of self-doubt and fear of being exposed as a fraud, which can further impact well-being and academic performance;^33^ suicidal ideation;^4^^,^^13^^,^^28^ and career regret.^13^

The concept of coping is defined as one’s attempts to deal with the internal and/or external demands of an encountered situation that is perceived as stressful.  Coping mechanisms and strategies are many and variable in nature.  Broader social, cultural and/or historical factors may impact the coping strategies used by a given individual; this is important, as the unique culture of medicine and medical practice may impact medical students’ willingness to admit weakness and/or seek assistance when confronted with stressors.^34^  Coping strategies used by medical students can be categorized as either problem-focused (dealing with and finding solutions to the demands of the stressor itself) or emotion-focused (using techniques to manage one’s internal emotional responses to the stressor.^10^  Problem focused strategies include seeking family, peer or mentor support;^35^^-^^37^ time management skills and spiritual beliefs/practices;^38^ or hobbies and relaxation exercises.^39^  Emotion-focused strategies include denial, avoidance or disengagement;^35^^, ^^40^ self-blame and withdrawal;^30^ or meditation, non-study activities and/or substance use.^20^  Studies have found differences concerning coping strategies between students in pre-clinical versus clinical portions of the medical school curriculum;^40^^,^^41^ and differences in coping strategies based on student gender.^42^

Efforts to mitigate these stressors have led to the implementation of wellness initiatives, resilience training, and peer-support programs in medical schools.^43^ While these interventions show promise, there remains a need to assess their effectiveness and refine approaches tailored to student needs.  One study suggested that medical students’ well-being benefits from structured programs that incorporate mindfulness, cognitive-behavioral strategies, and mentorship.^44^ A consistent theme of the literature is that medical schools should prepare students for facing the stressors associated with medical practice by providing them with deliberate education designed to enhance their coping skills.

Given the stress associated with the rigor of medical student education, we felt it worthwhile to examine two inter-related issues:  how and when do medical students experience stress, and how do they cope with it?  Accordingly, the purpose of our study was to examine both stressors and coping skills as reflected in our student population at a relatively new medical school located in the southeastern United States.  We were interested in identifying key points in time when students experienced various stressors within the four-year medical school curriculum, and ways in which our students coped with those stressors; was perceived stress associated with different coping strategies as they progressed through medical school?  We also wanted to determine whether stressors and coping mechanisms used by our students were different based on curricular phase (i.e., pre-clinical versus clinical) and student gender.  We hoped to use the results of our study to design interventions that would support our students at various points in time during their four-year journey through medical school.

## Methods

### Study Design and Participants

We designed a repeated cross-sectional cohort study to collect data at various time points from seven different medical student graduating classes (2016-2022).  For two of those classes, (2019 and 2020), we collected data yearly from students during each of their four years of medical school.  For these two classes, we were particularly interested in examining whether and how student stressors and coping skills changed during the course of their four-year medical school experience.  Our cross-sectional study involved cohort follow-up at the group, not individual, level.

### Instruments

To assess our students’ perception of their experiences of stress, students completed the Perceived Stress Scale (PSS), a widely used instrument consisting of ten items.^45^ It asks respondents to report the frequency of stress-related thoughts and feelings over the last month, using a 4-point Likert scale ranging from "almost never" to "very often."  A recent study^46^ found that the PSS had satisfactory internal consistency and reliability when validated with a population of 267 final year medical and health sciences students (Alpha = 0.79 to 0.86).  It was also used in a recent study that examined stress associated with the COVID-19 pandemic among US medical students.^47^ A systematic review of this scale found that it has acceptable psychometric properties but called for future studies to validate the scale among different study populations.^48^

Coping strategies were assessed using a modified version of the Coping Orientation to Problems Experienced (COPE) questionnaire.^49^^-^^50^ The original version of this instrument contained 60 items, and it has been modified to reflect fewer items in a variety of studies. The scale’s author recommended reducing the original 60-item scale to 28 items to address participant response burden and redundancy.^50^ Different versions of the COPE instrument have been validated in previous research involving health-related issues and subjects, with satisfactory reliability reported (Alpha** =** 0.50 to 0.90).^50^ The 28-item version of the scale contains 14 subscales consisting of two items, each of which reflects a different type of coping strategy.  For our study, we reduced the 28-item version to a 17-item version, selecting items from 13 of the 14 subscales; we also re-worded the items to reflect present instead of past tense.  We chose not to include either item from the subscale labeled “denial,” feeling that it wasn’t relevant to our student population.  Our approach to using fewer COPE items was consistent with published recommendations of the author of the COPE instrument, which invites researchers to modify the instrument to fit particular needs and populations.^50^

### Data Collection

Data collection occurred during the spring semesters of seven academic years (2015-2016 through 2021-2022).  A summary of all data collected by class is shown in Table 1.  We collected data during each of the four years of medical school for two of the seven classes of students (classes of 2019 and 2020).  This portion of the study is best described as a repeated cross-sectional cohort study involving four annual waves within two classes.  For these two classes, comparisons were made across academic years to assess potential changes in perceived stress and coping strategies over time.

**Table 1:** Study Participants – All Data Collection Years

Class	M1	M2	M3	M4	Total
2016	0	0	0	23	23
2017	0	0	27	30	57
2018	0	28	17	11	56
2019*	18	17	17	8	60
2020*	21	15	14	10	60
2021	16	12	0	0	28
2022	20	0	0	0	20
Total	75	72	75	82	304

*Two classes for which we collected data in repeated waves at various time points across four years of medical school

Participation was voluntary and all responses were anonymized. An honest broker de-identified the data before providing it to investigators to ensure confidentiality. We collected the data in two ways, depending on the availability of a given medical student class:  via an online survey, or in person via paper surveys.  This study was approved by our institutional review board (IRB) to ensure ethical compliance (Carilion Clinic IRB, March 1, 2016,study deemed exempt from further review). Informed consent was obtained from all participants, and confidentiality measures were maintained throughout the study.

### Data Analysis

For the Perceived Stress Scale, scoring is obtained by summing the points awarded to each item, and the 0 to 40 range is broken down into three levels of perceived stress (0-13 = low stress, 14-26 = moderate stress, and 27-40 = high stress).We report total scores on the PSS scale, with possible scores ranging from 0 to 40, with higher scores indicating higher perceived stress.

For the COPE questionnaire, items are scored based on a 4-point Likert scale ranging from "I haven’t been doing this at all" to "I have been doing this a lot.”  Scoring for the 28-item version is based on a range of 2 to 8 on each subscale, with higher scores on each subscale indicating greater use of that particular coping strategy.  We report scores on the COPE scale using item means and standard deviations.

In order to examine associations between perceived stress and coping skills, we converted all scale scores to *z*-scores for analyses performed on data pertaining to the two classes for which we collected data during each of their four years of medical school. We used the Pearson correlation procedure to examine these associations and report 95% confidence intervals for these data.

Additionally, we pooled data from all classes surveyed into two categories for additional analysis. We analyzed mean scores on the brief COPE scale based on both student sex and whether students were in pre-clinical versus clinical settings, using Welch’s independent samples t-test procedure (level of significance = 0.05).  Welch’s t-test was used to account for unequal sample sizes in the cohorts.  We also examined inter-item correlations for each instrument, and between the two instruments, using the Pearson correlation procedure including 95% confidence intervals.  Finally, we analyzed the two survey instruments using Cronbach’s Alpha reliability method and included all survey responses for both of the scales used.

In addition to the two ratings scale instruments, we asked our students to respond to an open-ended question asking them to identify three events or experiences that had caused stress during their current academic year.  The question was worded as follows: “as part of this project, we ask you to list up to three events/experiences that have caused you stress since the beginning of this academic year.”

For the two classes from which we collected data during each of their four years of medical school (2019 and 2020), we analyzed responses from this open-ended question using qualitative thematic analysis. Two independent reviewers coded the responses to identify recurring themes related to stressors experienced by medical students. Discrepancies in coding were resolved through discussion to ensure reliability.

## Results

We first report results for the two classes from which we collected data during each of their four years of medical school.We collected a total of 60 surveys from each of these two classes; the survey participation rate for the class of 2019 was 36.6% (60 of 164), and for the class of 2020 was 35.7% (60 of 168).  Different students responded to the surveys at each wave of data collection, with no evidence of the exact same students being followed; thus, our description of this portion of the study as a repeated cross-sectional cohort study involving four annual waves within two classes.

For the PSS, across all four study years, the mean score for the class of 2019 was 23.0 (SD = 2.51), and for the class of 2020 was also 23.0 (SD = 2.88).  PSS scores for the class of 2019 ranged from a low of 19.0 to a high of 30.0.  By year, the mean PSS scores for the class of 2019 were 24.2 (SD = 2.65) during year one, 23.5 (SD = 2.38) during year two, 24.1 (SD = 2.36) during year three and 23.4 (SD = 2.59) during year four.  PSS scores for the class of 2020 ranged from a low of 18.0 to a high of 31.0.  By year, the mean PSS scores for the class of 2020 were 22.7 (SD = 2.71) during year one, 23.8 (SD = 2.77) during year two, 23.8 (SD = 3.10) during year three and 25.0 (SD = 2.37) during year four.

**Figure 1:**
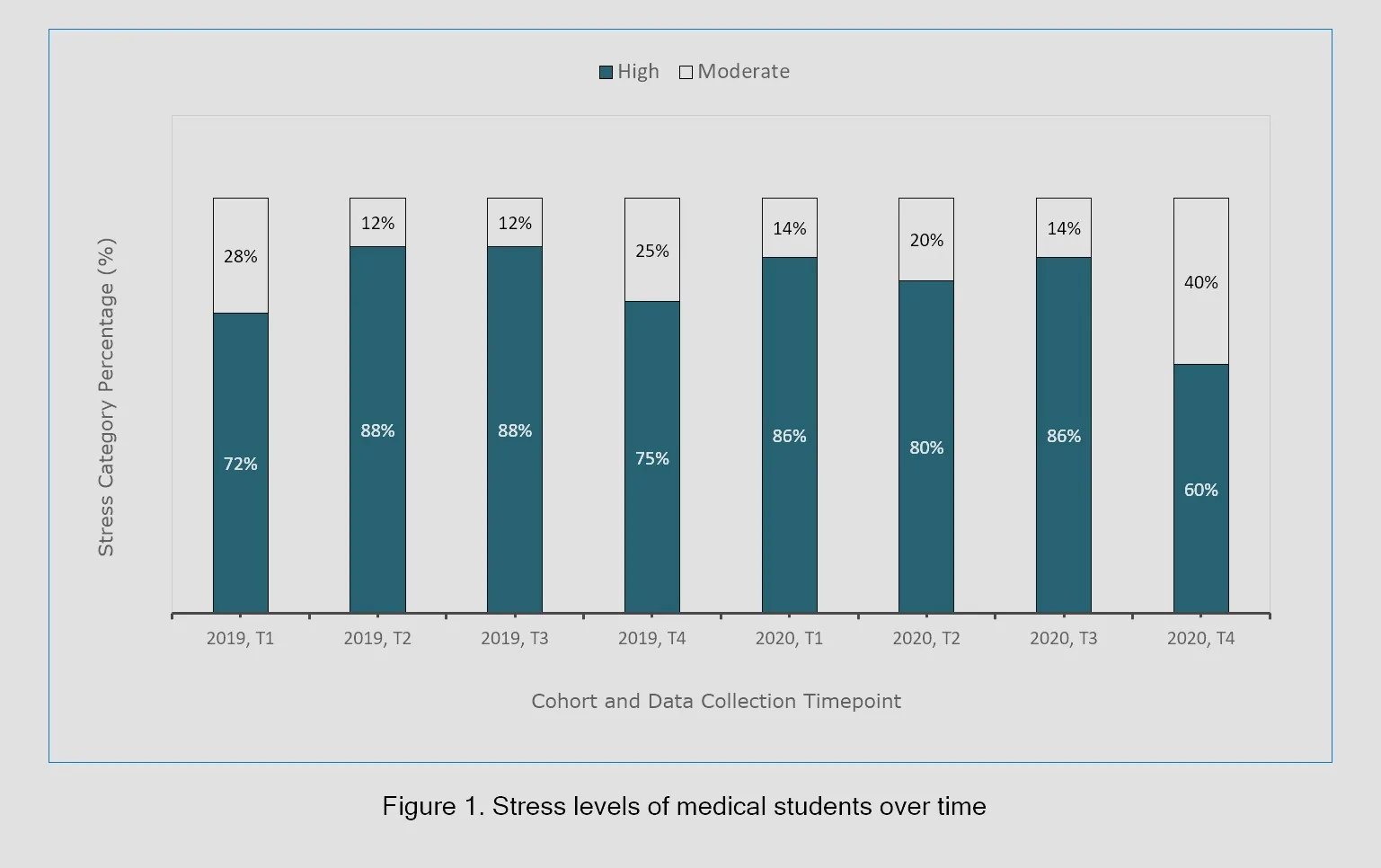
Stress levels of medical students over time

**Figure 2:**
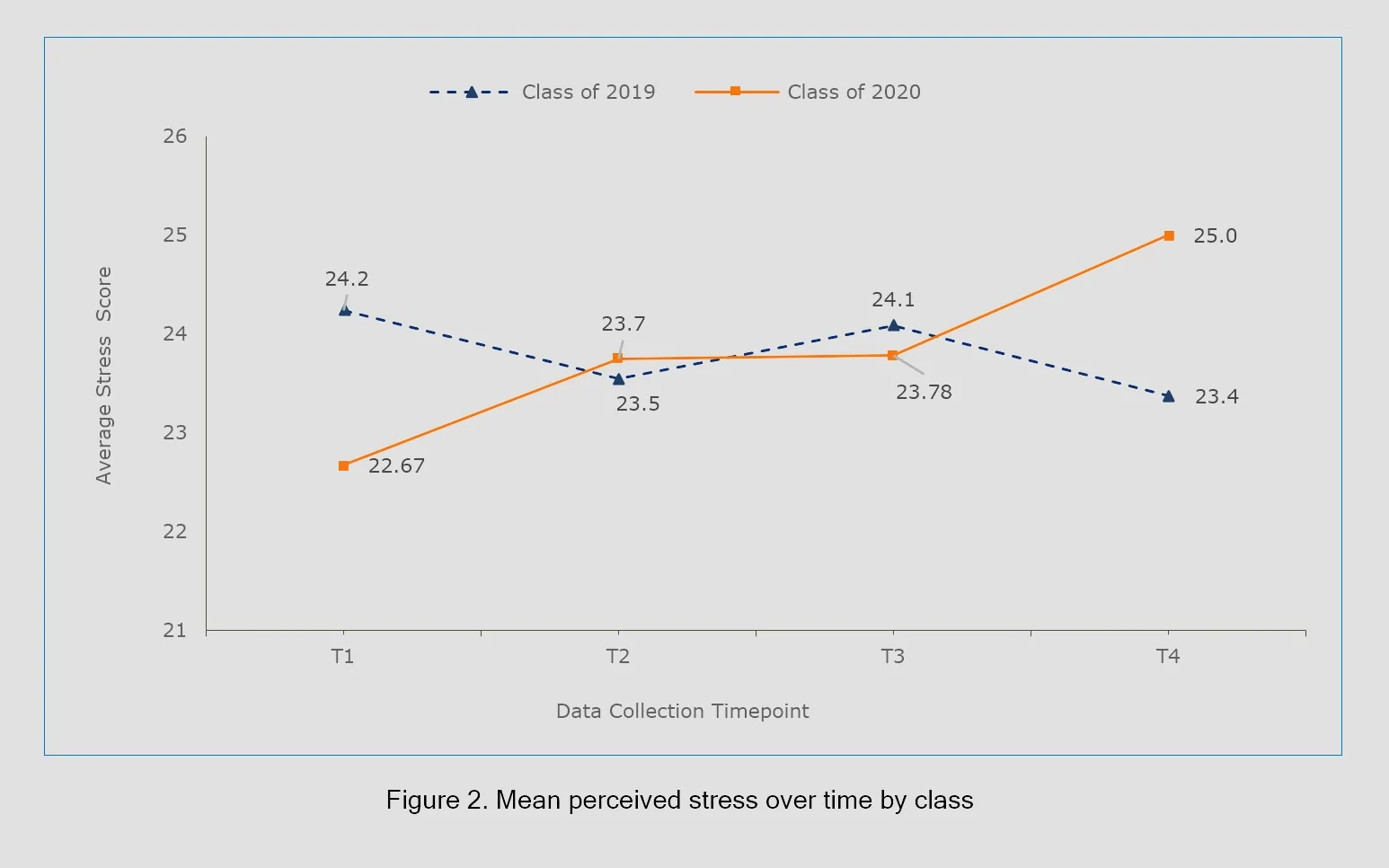
Mean perceived stress over time by class

All mean scores on the PSS for these two classes are considered to be in the moderate stress category (score range of 14-26).  However, the range of scores for both classes indicates that some students fell into both the moderate (14-26) and high (27-40) stress categories at various points in time.  Of note, at no time did any student PSS score fall into the low stress (0-13) category.  Figure 1 displays categorical stress levels over time (i.e., moderate and high) for each class.  PSS mean score trajectories for the two classes were noticeably different, as shown in Figure 2.  Mean score patterns for perceived stress tend to show more spread at time one/first year of medical school and time four/fourth year of medical school. For the class of 2019, mean scores fluctuated in a more upward and downward pattern, whereas for the class of 2020, mean scores increased gradually over time.

For the brief COPE scale, in Table 2 we report mean scores and standard deviations for each of the seventeen ratings items and at each time point, for both the class of 2019 and class of 2020.  The score patterns for the two classes are very similar.  The lowest mean score across both classes was for item 4, “I give up trying to deal with the stress” (M = 1.1, SD = 0.35) by M4 students in the class of 2019; the highest mean score across both classes was item 3, “I get emotional support from others” (M = 3.8, SD = 0.46), also by M4 students in the class of 2019.

To examine associations between perceived stress and coping skills, we converted all scale scores to *z*-scores and analyzed the data based on both classes combined.  We found two significant correlations between perceived stress and coping skills.  A higher use of the coping mechanism of “venting” was positively associated with higher perceived stress levels, *r *= 0.24, p = 0.01, 95% CI [0.06, 0.40].  And, a higher use of the coping mechanism of “positive reframing” was negatively associated with lower perceived stress levels, *r *= -0.19, p = 0.05, 95% CI [-0.36, -0.01].

We also examined the association between perceived stress and coping skills at the four time points of data collection, i.e., during M1 through M4 years.  We found that the coping skill of humor (“I make jokes about the stress”) was negatively correlated with perceived stress by M2 students, *r *= 0.33, p = 0.045, 95% CI [0.16, 0.48], but was positively correlated with perceived stress by M4 students, *r *= 0.45, p = 0.10, 95% CI [0.29, 0.58].  Thus, the role of humor as a coping strategy appeared to shift over time. In second-year students, humor was negatively associated with perceived stress, potentially serving as a light-hearted buffer in a didactically dense and cognitively demanding year. However, by the fourth year, humor was positively associated with higher stress levels, possibly indicating its use as a defensive or compensatory mechanism in the face of escalating uncertainty surrounding residency placement and professional identity formation.

**Table 2:** Modified Brief COPE Scale – Two Medical Student Classes

#	Item Category	Class of 2019Mean and Standard Deviation				Class of 2020Mean and Standard Deviation			
		M1	M2	M3	M4	M1	M2	M3	M4
1	Self-distraction	M=2.9 SD=0.73	M=2.8 SD=0.73	M=2.8 SD=0.75	M=3.1 SD=1.25	M=2.6 SD=0.76	M=2.9 SD=1.06	M=2.8 SD=0.89	M=3.0 SD=0.82
2	Active coping	M=3.0 SD=0.91	M=2.9 SD=0.66	M=2.9 SD=0.93	M=3.0 SD=0.53	M=2.8 SD=0.85	M=2.9 SD=0.59	M=3.0 SD=0.96	M=2.9 SD=0.88
3	Emotional support	M=2.7 SD=0.84	M=2.8 SD=0.95	M=2.9 SD=0.81	M=3.8 SD=0.46	M=2.4 SD=0.94	M=2.8 SD=1.15	M=2.8 SD=0.97	M=2.8 SD=0.79
4	Behavioral disengagement	M=1.4 SD=0.61	M=1.4 SD=0.80	M=1.5 SD=0.63	M=1.1 SD=0.35	M=1.7 SD=1.08	M=1.5 SD=0.52	M=1.9 SD=0.77	M=1.5 SD=0.71
5	Active coping	M=3.4 SD=0.78	M=3.3 SD=0.77	M=3.3 SD=0.86	M=3.5 SD=0.53	M=3.2 SD=0.83	M=3.1 SD=0.74	M=3.1 SD=0.62	M=3.3 SD=0.67
6	Substance use	M=1.2 SD=0.43	M=1.5 SD=0.62	M=1.6 SD=0.72	M=1.5 SD=1.07	M=1.3 SD=0.55	M=1.4 SD=0.63	M=1.6 SD=0.76	M=1.4 SD=0.84
7	Positive reframing	M=2.1 SD=0.90	M=2.1 SD=0.99	M=2.4 SD=1.02	M=2.8 SD=1.16	M=2.3 SD=0.98	M=2.3 SD=1.18	M=2.6 SD=0.84	M=2.5 SD=0.97
8	Self-blame	M=3.1 SD=0.76	M=3.4 SD=0.70	M=3.3 SD=0.86	M=2.8 SD=1.39	M=3.1 SD=1.02	M=2.9 SD=0.7	M=3.1 SD=0.86	M=3.2 SD=0.92
9	Planning	M=3.1 SD=1.00	M=2.7 SD=0.77	M=2.8 SD=0.86	M=3.3 SD=0.71	M=2.8 SD=0.89	M=2.7 SD=1.03	M=2.9 SD=0.66	M=3.0 SD=1.15
10	Emotional support	M=3.1 SD=0.76	M=2.7 SD=0.99	M=3.0 SD=0.82	M=3.3 SD=0.89	M=2.6 SD=1.05)	M=2.8 SD=1.01	M=2.8 SD=1.19	M=3.1 SD=0.88
11	Humor	M=2.6 SD=1.20	M=2.6 SD=0.79	M=2.5 SD=0.89	M=2.5 SD=1.20	M=3.0 SD=0.83	M=3.1 SD=0.88	M=3.1 SD=0.83	M=3.3 SD=1.06
12	Self-distraction	M=3.3 SD=0.69	M=3.0 SD=0.71	M=3.1 SD=0.77	M=3.1 SD=0.83	M=2.9 SD=0.72	M=3.0 SD=0.76	M=2.6 SD=1.16	M=3.2 SD=0.79
13	Venting	M=2.5 SD=0.92	M=2.4 SD=0.80	M=2.8 SD=0.68	M=2.9 SD=0.99	M=2.5 SD=0.83	M=2.4 SD=0.83	M=2.4 SD=0.74	M=2.6 SD=0.97
14	Religion	M=1.7 SD=1.02	M=1.5 SD=0.87	M=1.5 SD=0.89	M=1.8 SD=0.89	M=1.5 SD=0.83	M=1.9 SD=1.13	M=2.1 SD=1.21	M=1.8 SD=1.23
15	Instrumental support	M=2.2 SD=1.10	M=2.7 SD=1.05	M=2.6 SD=0.72	M=2.9 SD=0.99	M=2.0 SD=0.69	M=2.7 SD=1.11	M=2.4 SD=0.84	M=2.8 SD=0.92
16	Acceptance	M=3.1 SD=0.87	M=3.0 SD=0.79	M=2.6 SD=0.72	M=2.6 SD=0.92	M=3.1 SD=0.79	M=2.9 SD=0.8	M=2.9 SD=0.62	M=2.8 SD=0.63
17	Planning	M=3.2 SD=0.73	M=3.3 SD=0.85	M=2.9 SD=0.81	M=3.0 SD=0.76	M=2.9 SD=0.85	M=2.9 SD=0.96	M=2.9 SD=1.00	M=3.0 SD=0.94

Item key: 1 I turn to work or other activities to take my mind off things 2 I concentrate my efforts on doing something about the stressful situation I am in 3 I get emotional support from others 4 I give up trying to deal with the stress 5 I take action to try to make the situation better 6 I use alcohol or other drugs to help me get through the stress 7 I try to see my stress in a different light, to make it seem more positive 8 I am critical of myself 9 I try to come up with a strategy about what to do about my stress 10 I get comfort and understanding from someone 11 I make jokes about the stress 12 I do something to think about it less, such as going to the movies, watching TV, reading, daydreaming, sleeping or shopping 13 I express my negative feelings 14 I try to find comfort in my religion or spiritual beliefs 15 I try to get advice or help from other people about what to do 16 I try to learn to live with it. 17 I think hard about what steps I should take next

The coping skill of positive reframing (“I try to see my stress in a different light, to make it seem more positive”) was negatively correlated with perceived stress by M2 students; *r *= -0.33, p = 0.04, 95% CI [-0.48, -0.16].  Three other areas of coping skills were also positively correlated with perceived stress by M4 students: planning (“I try to come up with a strategy about what to do about my stress”); *r *= 0.44, p= 0.01, 95% CI [0.28, 0.57]; self-blame (“I am critical of myself”); *r *= 0.36, p= 0.04, 95% CI [0.19, 0.51]; and venting (“I express my negative feelings”); *r *= 0.47, p = 0.007, 95% CI [0.32, 0.60].  We found no significant correlations between perceived stress and coping skills for M1 students or M3 students.

Our analysis of qualitative data from these two classes illuminated specific contextual stressors that students face during each phase of training.  These analyses revealed several themes across all 4 medical school years that demonstrated year-specific and general patterns. These themes were academic stress, residency application and the match process, and personal life issues.

Academic stress was a constant in all years and included standardized tests, block exams, the amount of academic material, and pressure to perform. Spikes in perceived stress during the M2 and M4 years corresponded with preparation for high-stakes exams (e.g., Step 1 and Step 2) and the pressures of the residency match process. These findings suggest that students’ coping strategies may be directly shaped by the nature of the stressors encountered at each stage.  M1s and M2s emphasized adjusting to medical school rigor and striving for a passing score on USMLE Step 1, while M3s identified shelf exams and clinical performance as greater stressors.

M4s identified both the academic pressures of USMLE Step 2 and final rotations. Anxiety around remediation, failing grades, and competition with classmates was also widespread.

The residency application and match processes were the most prevalent stress-related factor in M3 and M4 students. Preparing and applying for residency and attending interviews was a considerable source of anxiety. Match Day was a common point of stress for M4s. Students also described the stressors as application logistics of ERAS, away rotations, traveling, and rank list submission that were associated with the residency process. Students felt emotional and financial stress attributed to traveling for residency interviews, as well as stress related to couples matching.

Students across all class years mentioned how personal life issues (e.g., relationships, family, mental/physical health, or logistical stressors) outside of medical school negatively impacted their academic and professional performance. M1 students described adjustment difficulty to a new social environment and being distanced from family. M2 students reported feeling stressed by the need to balance academic workload and major life events. M3 students and M4 students described frayed relationships, death/illness in the family, financial burden, and burnout as stressors.

Student comments were also examined for themes based on curricular year, i.e., pre-clinical versus clinical portions of the curriculum (see Table 3).  During the preclinical years, academic testing and assessments were the most frequently mentioned stressor among students. Additionally, preclinical students reported stress related to balancing the academic rigor of medical school with personal relationships and family. Preparation for USMLE Step 1 was a dominant stressor during the M2 year. This is represented by comments such as “studying for step” and “step (general)” that were among the most frequent codes in M2’s lists of stress. In the clinical years, there was a clear shift in the stressors from the more academic to the preparation for careers and performance in the clinical arena. M3 students were worried about USMLE Step 2, clerkship subject exams, and keeping up their grades in their rotations. As M4s are close to graduation, their main stressors were interviews, away rotations and the residency match, a combination of academic, professional, and personal concerns.

Comparisons of stressors and preferred coping mechanisms by gender revealed some differences.  Male students were more likely to list USMLE Step exam preparation, studying, and research as stressors whereas female students reported a broader array of interpersonal and academic stressors. Despite these differences, USMLE Step exams and other evaluations were clearly the prevailing theme for both males and females. Additionally, male students were more likely to use humor, acceptance, and positive reframing as ways to cope with stressors, particularly in the preclinical years.

Based on our literature review, we wanted to examine whether there were differences in both perceived stress and coping skills based on two categories:  students based on sex (male/female) and students in pre-clinical versus clinical settings.  For these analyses, we included data from all student participants across all classes of students participating in the study (total of 304 surveys).

**Table 3:** Themes of Students’ Qualitative Comments by Curricular Year (Classes of 2019 and 2020)

Medical School Year	Stressors		
M1	Adjusting tomedical school learning	Final exams and blockexams	Establishingsocial groups and separation from family
M2	Studying forUSMLE Step 1	Waiting forUSMLE Step 1 score	Managinglong-distancerelationship
M3	Shelf exams	Clinical rotation performance	Applying to away rotations
M4	Residencyapplication and match process	Planning forinternship and relocation	Couples match and relationship planning

PSS scores for female students ranged from a low of 15.4 to a high of 33.4; for male students, the PSS scores ranged from a low of 15.9 to a high of 31.4.  Mean PSS scores across all four years were 23.0 (SD = 2.31) for male students and 23.5 (SD = 2.82) for female students; there were no statistically significant differences on mean PSS scores based on student sex, at any of the four data collection time points (years M1-M4).

For the brief COPE scale, the analysis based on student sex showed that certain coping skills were more likely to be used at various points in time.  Male students were more likely than female students to use humor (“I make jokes about the stress”); *z* = 0.3 males, 95% CI [-1.66, 2.26]; -1.1 females, 95% CI [-3.06, 0.86]; p = 0.02, *d* = 1.40 as a coping skill during the M1 year, and were also more likely to use positive reframing (“I try to see my stress in a different light, to make it seem more positive”); *z* = 0.17 males, 95% CI [-1.79, 2.13]; -0.48 females, 95% CI [-2.44, 1.48]; p = 0.005, *d* = 0.65 and acceptance (“I try to learn to live with it”); *z* = 0.44 males, 95% CI [-1.52, 2.40]; -0.48 females, 95% CI [-2.44, 1.48]; p = 0.03, *d* = 0.92 as coping skills during the M2 year.  We found no significant differences in coping skills based on student sex during year three. For the M4 year, we found that male students were more likely to use acceptance (“I try to learn to live with it”); *z *= 0.22 males, 95% CI [-1.74, 2.18]; 0.43 females, 95% CI [-2.39, 1.53]; p = 0.023, *d* = 0.65 as a coping skill than female students.  Finally, there were no differences in coping skills based on pre-clinical versus clinical settings.

Reliability analyses of the two ratings scales were computed based on pooling all completed surveys across all years of the study.  These analyses revealed that both the Perceived Stress Scale (PSS) (Alpha = 0.86) and the Brief COPE Scale (Alpha = 0.71) had acceptable levels of reliability.  The results of scale reliability are consistent with prior literature concerning the use of these scales with medical student populations.

## Discussion

This study aimed to identify evolving stress and coping experiences throughout the four years of medical school experience.  While there are some studies that have examined stress among first year medical students,^51^^-^^52^ we could find only three studies that examined medical student stressors in a similar fashion across multiple years of medical school.  One study^5^ tracked 377 German medical students over six years, using standardized tools to examine anxiety, stress, burnout, depression and coping strategies, demonstrating changes in stressors over time and higher vulnerability to stressors by female students.  Another study^22^ surveyed 297 German medical students over three years, with a focus on how student stress impacted career ambitions.  And, a third study^25^ examined the association between burnout, resilience, and life satisfaction among 190 medical students over a 20-month period.

Consistent with findings from these studies, our study provides additional supporting evidence that the four-year medical school process is an experience that involves significant stress and requires the ability to effectively cope with that stress.  It underscores the chronic nature of stress in medical education and highlights the dynamic relationship between perceived stress and coping strategies across the four-year curriculum. Our findings support a paradigm shift in which stress is not viewed solely as an early curricular issue but as a persistent challenge requiring continuous support. Our students reported moderate-to-high perceived stress throughout their educational journey.  Consistent with other studies,^4^^-^^5^ perceived stress by our students was moderate and relatively stable across the study period, yet also involved fluctuations in stress levels at various moments in time. There was a slight variation in patterns of stress across classes, with the class of 2020 demonstrating a more gradual increasing pattern over time. This may reflect the cumulative nature of academic, clinical, and career-planning stressors increasing over time for that cohort. For the class of 2019, there was more variability over time, perhaps highlighting differences in experiences across cohorts or external factors specific to the class of 2019. These patterns may reflect variability attributable to the class or external factors such as the academic calendar or institutional factors. Regardless, these patterns remind medical schools that stress is not a first-year phenomenon that will dissipate over time, but rather a chronic stress that must be addressed at all stages of medical school education. Early intervention is critical, as patterns of stress begin in the preclinical years and may intensify if left unaddressed.

An important contribution of the study is its analysis of coping strategies and their relationships to perceived stress. A key finding is that different types of coping strategies tend to vary significantly among students. Positive reframing was significantly associated with lower perceived stress, suggesting that it might be a protective factor, which should be strengthened through medical curricula. In contrast, venting and self-blame correlated with higher scored levels of perceived stress, particularly among fourth-year students. While some students tend to use adaptive, problem-focused coping strategies, others seem to rely on emotion-focused responses that could further exacerbate their stress levels.

Interestingly, the function of humor as a coping mechanism appeared to evolve over time, such that humor was associated with lower stress in second-year students, possibly serving as a buffer in a didactically dense year, but with higher stress in fourth-year students, perhaps reflecting its use as a defense mechanism in response to escalating uncertainty surrounding career decisions and residency placement. Gender differences in dealing with stress were also a prominent finding of our study, with male students tending to use acceptance, positive reframing, and humor to greater effect. This was true especially in pre-clinical years. Interestingly, the differences in coping were not associated with differences in perceived stress, which could indicate that male and female students have the same levels of stress but just cope with it differently, with important implications for developing personalized interventions. For many students, especially those far from home or with limited local support, feelings of isolation can amplify the stress of academic and clinical demands.

Our qualitative analysis of student-reported stressors reflected skills and challenges previously reported in the literature. Principal stressors were based on a combination of academic (volume of material and time pressure, transition to clinics) and interpersonal (social comparison) factors in medical education. These findings highlight the pressing need for early support systems that can buffer students from the compounding effects of stress before it becomes established, and also a need for more personalized, gender-sensitive wellness programming that acknowledges differences in how students experience and manage stress.  This analysis also highlights the importance of personalized support strategies that change as students progress through medical school and adapt to gender-specific stressors.

### Limitations

There are several important limitations to our study that must be considered. First, participation was voluntary and response rates were modest at best; and, because our study was cross-sectional in nature, it cannot establish causality and is susceptible to selection and recall bias.  Students under extreme stress or profoundly disengaged with their school experience may have been under-represented in the study, and this could have impacted our results. Second, although the COPE scale was reduced in size to limit the burden on participants, this shortening may have resulted in the exclusion of relevant coping dimensions, particularly those relating to avoidance and denial. Third, we were not able to collect meaningful numbers of completed surveys for more than two classes of medical students, due to logistical and related considerations; and, for these two classes, response rates were lower (36%, 35%) than would be considered optimal for survey-based research.  Finally, this was a single-institution study which may inhibit generalizability; however, our findings are largely reflective of existing literature.

Strengths of the study include the opportunity to collect data over an extended time period from several medical student classes, and the use of validated instruments carefully adapted for medical student populations. Internal reliability of the PSS and brief COPE scales were consistent over multiple years, underscoring the robustness of these results. Finally, the use of qualitative data allowed a deeper understanding of the students’ lived experiences not otherwise captured by purely quantitative measures.

## Conclusions

In light of these findings, we recommend that medical schools develop tailored interventions targeting both adaptive coping strategies and stress-reduction techniques throughout the four-year curriculum. Structured support systems such as mentorship programs, peer groups, and training in cognitive-behavioral techniques may promote more effective coping. Additionally, proactive measures during known transition points, such as entering clinical rotations or applying to residency, may help mitigate stress surges. Future research should explore how institutional culture, curricular structures, and psychological safety influence students’ willingness to seek support and employ adaptive coping mechanisms. Partnering with students to co-design these interventions may further ensure their relevance, uptake, and long-term impact.

### Acknowledgments

We thank the medical students who participated in this study. The study received no internal or external grant support.

### Conflict of Interest

The authors declare that there is no conflict of interest.
